# PARTNERING WITH NUTRITION SERVICES PROGRAM PROVIDERS TO DISSEMINATE EVIDENCE-BASED PROGRAMS USING TELE-HEALTH

**DOI:** 10.1093/geroni/igz038.836

**Published:** 2019-11-08

**Authors:** Jeannette Beasley, Judith Wylie-Rosett, Mary Ann Sevick, Laura DeLuca, Joshua Chodosh

**Affiliations:** 1 New York University Langone Health, New York, New York, United States; 2 Albert Einstein College of Medicine, Bronx, New York, United States; 3 Yeshiva University, Bronx, New York, United States

## Abstract

Among adults ≥ age 65, 48% have prediabetes and are eligible to participate in the Medicare-covered Diabetes Prevention Program (DPP). We conducted a six-week pilot study to evaluate the feasibility and acceptability of a telehealth-adapted DPP for Nutrition Services Program (NSP) older adult meal program recipients. We enrolled NSP recipients (n=16) from a New York City senior center. These DPP participants attended weekly interactive DPP webinars and completed questionnaires covering lifestyle, physical activity, quality of life, and food records, and wore physical activity trackers. Retention was 75%; attendance averaged 80%; and weight loss was 2.9% (p=0.001). Our six-week pilot data suggest that a tele-adapted DPP intervention can achieve the Medicare reimbursement goals for attendance and 5% weight loss. We are surveying NSP recipients, who receive home-delivered meals, to evaluate the acceptability and feasibility of conducting a larger scale tele-adapted DPP intervention trial among NSP participants.

